# Enhanced dechlorination and biodegradation of 2-chloroaniline by a 2-aminoanthraquinone-graphene oxide composite under anaerobic conditions

**DOI:** 10.1038/s41598-019-48904-9

**Published:** 2019-08-26

**Authors:** Hong Lu, Tiantian Zhang, Yang Zhou, Jiti Zhou, Jing Wang, Xiaolei Wang

**Affiliations:** Key Laboratory of Industrial Ecology and Environmental Engineering, Ministry of Education, School of Environmental Science and Technology, Dalian University of Technology, Dalian, 116024 China

**Keywords:** Environmental biotechnology, Biocatalysis

## Abstract

The effect of a 2-aminoanthraquinone-graphene oxide (AQ-GO) composite on the anaerobic dechlorination and degradation of chloroanilines by an enriched bacterial consortium was investigated. The results showed that the maximal degradation efficiency of 20 mg/L 2-chloroaniline (2-CA) reached 91.4% at a dose of 20 mg/L AQ-GO in 30 d. Moreover, the pseudo-first-order rate constant of 2-CA degradation in the AQ-GO-mediated system was 2.9-fold higher than those in AQ- and GO-mediated systems alone. During this process, a synergetic effect between AQ and GO was observed, which was attributed to the increased intracellular and extracellular electron transfer pathways. GC-MS analysis showed that 2-CA could be degraded to hexanoic acid and ultimately mineralized to CO_2_. Illumina MiSeq sequencing revealed that additional AQ-GO significantly increased the relative abundance of *Firmicutes*. Further analysis showed that the populations of the genera *Oscillospira*, unclassified *Lactobacillales*, unclassified *Veillonellaceae* and *Ruminococcus* exhibited positive correlations with the rate constant of 2-CA degradation and the dehydrogenase activity of bacterial consortium. These findings indicated that AQ-GO promoted the enrichment of functional bacteria and increased the bacterial activity, resulting in the enhanced dechlorination and degradation of 2-chloroaniline.

## Introduction

Chloroanilines are widely used for the industrial synthesis of pesticides, plastics and dyes^1,2^. Thus, chloroanilines may be released into the environment directly during transportation and industrial processing or indirectly from the anaerobic biotransformation of many pesticides and dyes^2,3^. It is well known that the dechlorination and subsequent degradation of chloroanilines are slow processes under anaerobic conditions^2,4^. In particular, anilines with low chlorine content have lower abilities to obtain electrons for dechlorination than polychlorinated anilines, indicating that di- and mono-chloroanilines are difficult to dechlorinate and further degrade under anaerobic conditions.

Recently, graphene oxide (GO), as an emerging kind of nanomaterial, has received increasing attention owing to its large surface area, good mechanical strength and relative biocompatibility^5^. Some studies have shown that reduced GO (rGO) can be used as an electron mediator to increase the chemical and microbial reduction of azo dyes and nitroaromatics^6–8^. Moreover, Wang *et al*. (2014) found that a composite of GO and anaerobic sludge presented good settling performance when GO was added to an anaerobic sludge system^8^. This observation was attributed to the effect of some oxygen-containing functional groups of GO, which include alcohols, epoxides, and carboxylic acids. Based on these functional groups, GO could be modified to improve its electron mediator performance. It has been well demonstrated that 2-amino-3-chloro-1,4-naphthoquinone- and anthraquinone-2-sulfonate-modified GO exhibited better electron mediator performance in Cr(VI) reduction and azo dye decolorization than GO^9,10^. Moreover, it has been reported that anthraquinone compounds (ACs) can be used not only as redox mediators to accelerate electron transfer for dechlorination^11,12^ but also as electron acceptors to improve the degradation rates of electron donors, including phenol^13^. Accordingly, AC-modified GO is hypothesized to exhibit better electron mediator performance for the reduction of electron acceptors and the degradation of electron donors than GO and ACs alone.

In the present study, GO was modified by 2-aminoanthraquinone (AQ) using a one-step chemical method. 2-Chloroaniline (2-CA) was used as a model pollutant, and the acceleration effect of the AQ-GO composite on the anaerobic dechlorination and degradation of 2-CA was investigated in detail. To better understand the acceleration effect of AQ-GO on 2-CA degradation, the dynamics of the enriched bacterial consortium were analysed using Illumina MiSeq sequencing.

## Results and Discussion

### Characterization of the AQ-GO composite

**Figure 1:**
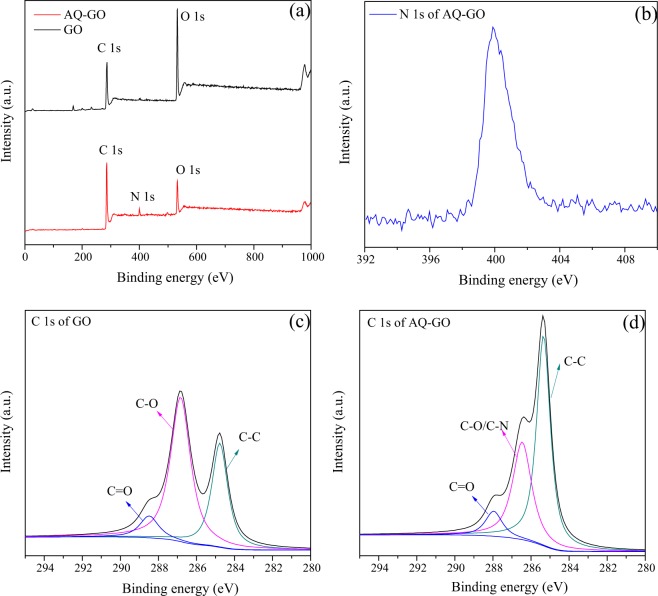
XPS spectra of GO, AQ-GO (**a**), N 1s of AQ-GO (**b**), C 1s of GO (**c**) and C 1s of AQ-GO (**d**).

The AQ-GO composite was successfully prepared via a one-step chemical reaction (SI Fig. 1), as confirmed by the appearance of the characteristic absorption bands of -CONH at 1671 cm^−1^ (C=O stretching), 1587 cm^−1^ (N-H deformation) and 1336 cm^−1^ (C-N stretching) in the infrared spectrum of the AQ-GO composite (SI Fig. 2). Further analysis showed that an N 1s peak at 400 eV appeared in the X-ray photoelectron spectrum of AQ-GO (Fig. 1). Moreover, compared with the C 1s spectrum of GO, it was also observed that the intensity of the C-C peak increased and the intensity of the C-O peak decreased in the C 1s spectrum of AQ-GO, indicating that GO was partially reduced during AQ-GO preparation. It was also confirmed by X-ray diffraction (XRD). As shown in SI Fig. 3, compared with the XRD pattern of GO with a d-spacing of 0.87 nm, the 10.5° peak partially decreased and a broad peak starting from 16.7° to 27.5° appeared in the XRD pattern of the AQ-GO. The partial interlayer spacing of AQ-GO decreased to 0.43 nm, suggesting that the oxygen-containing functional groups in AQ-GO was only partially removed. The electrical conductivity of AQ-GO was detected using electrochemical method. As shown in SI Fig. 4, when AQ-GO-modified GCE was used, the redox peaks could be remarkably observed, indicating that AQ-GO is capable of transferring electrons. However, the mixture of AQ and GO has not electrical conductivity. Based on X-ray photoelectron spectroscopy (XPS) analysis, the immobilization efficiency of AQ was approximately 4.3 mmol AQ g ^−1^ GO. This immobilization efficiency was higher than that in our previous study (2.7 mmol AQ g ^−1^ GO) due to the higher content of oxygen (43.2%) in the GO used in the present study compared with that (29.5%) in GO previously prepared by Zhang *et al*.^9^. Scanning electron microscopy (SEM) analysis was conducted to characterize the microstructures of GO and AQ-GO. As shown in SI Fig. 5, the surface of the AQ-GO sheets was slightly rougher than that of the GO sheets, indicating that the reaction conditions were mild during AQ-GO preparation.

### Effect of AQ-GO composite concentration on 2-chloroaniline degradation

**Figure 2:**
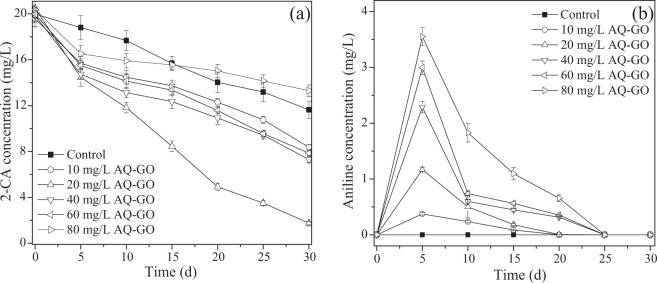
Effect of AQ-GO concentration on the dechlorination and degradation of 2-CA in 30 d. (**a**) 2-CA degradation; (**b**) aniline formation and degradation. Error bars show one standard deviation.

It has been reported that GO has cytotoxicity, which is caused by the direct contact between cells and the sharp edges of GO^14^. Moreover, high concentrations of ACs are toxic to cells. Accordingly, the effect of AQ-GO concentration on 2-CA degradation was investigated over 30 days. Figure 2a shows that AQ-GO could promote the degradation of 20 mg/L 2-CA when the AQ-GO concentration ranged from 10 mg/L to 60 mg/L. However, 80 mg/L AQ-GO resulted in a decreased degradation rate of 2-CA. The maximal degradation percentage of 2-CA (91.4%) was observed at a dose of 20 mg/L AQ-GO in 30 days. It seemed that the use of a suitable AQ-GO dose could enhance the degradation rate of 2-CA. During this process, the intermediate aniline was detected (Fig. 2b), indicating that the dechlorination process occurred. For the control system without AQ-GO, the concentration of the produced aniline was below the detection limit. AQ-GO addition resulted in aniline accumulation, indicating that AQ-GO could more significantly promote the dechlorination of 2-CA than aniline degradation.

Dehydrogenase activity (DHA) can serve as a good indicator of microbial activities (particularly electron transfer activity) during the anaerobic degradation of organic compounds^8^. Among the tested AQ-GO-mediated systems, the DHA of the bacterial consortium in the 20 mg/L AQ-GO-mediated system was the highest (SI Fig. 6). This result indicated that the stimulating effect of 20 mg/L AQ-GO on the electron transfer activity of the bacterial consortium was the most significant, which was consistent with the highest degradation rate of 2-CA in the presence of 20 mg/L AQ-GO.

The extracellular polymeric substances (EPS) of the bacterial consortia in the above reaction systems were also analysed after the reaction was over. The results showed that the change tendency in the concentration of EPS secreted by the bacterial consortia supplemented with 10–80 mg/L AQ-GO was consistent with that of the 2-CA degradation rate. As shown in SI Fig. 7, 20 mg/L AQ-GO resulted in the excretion of the largest amount of proteins, polysaccharides and total EPS. Previous studies have shown that EPS can bridge two neighbouring cells with each other and that GO can be used as a scaffold for cell attachment^15,16^; these processes are beneficial for interspecies electron transfer.

### AQ-GO-mediated degradation of chloroanilines

**Figure 3:**
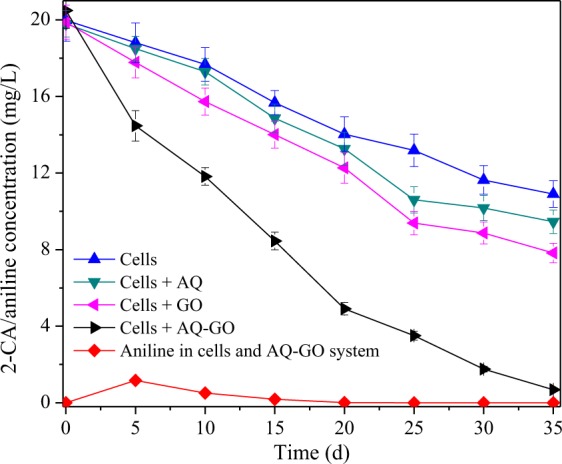
Time course of 2-CA biodegradation in different reaction systems. Error bars show one standard deviation.

Based on the above studies, 20 mg/L AQ-GO was used for 2-CA degradation and compared with 20 mg/L AQ and 20 mg/L GO alone. The results showed that less than 6% 2-CA was removed in the control systems with heat-killed bacterial consortia or without bacterial consortia in 35 days (data not shown), indicating that the effect of 2-CA adsorption on its degradation could be neglected. Figure 3 shows that 2-CA degradation followed a pseudo-first-order reaction and that its degradation rate in the reaction system without mediators was low (0.43 d^−1^, R^2^ = 0.99). Additional AQ (0.55 d^−1^, R^2^ = 0.97) and GO (0.67 d^−1^, R^2^ = 0.98) increased the degradation rate of 2-CA. Moreover, GO exhibited a better accelerating effect than AQ. It has been reported that GO can be used as scaffold for cell attachment^16^ and that rGO could be used as an electron mediator^7^; these processes are beneficial for transferring electrons from cells to 2-CA and between cells. AQ-GO addition resulted in a significantly increased degradation rate of 2-CA (1.97 d^−1^, R^2^ = 0.93), which is over 2.9-fold higher than those in AQ- and GO-mediated systems alone. During this process, a low concentration of aniline was detected. Moreover, AQ-GO accelerated the degradation of aniline (SI Fig. 8). In fact, in AQ-GO-mediated reaction system, AQ-GO was reduced by the bacterial consortium, which resulted in the better catalytic activity of reduced AQ-GO compared with that of AQ-GO (SI Fig. 9). These studies indicated that AQ-GO and bioreduced AQ-GO could be used as electron mediators to accelerate the dechlorination and degradation of 2-CA. Further analysis found that the pseudo-first-order rate constant of 2-CA degradation in the AQ-GO-supplemented system was higher than the sum of those in the AQ- and GO-supplemented systems, implying the existence of a synergistic effect between AQ and GO. Our previous study showed that the synergistic effect between anthraquinone-2-sulfonate (AQS) and GO was attributed to electron transfer from AQS to rGO, either directly or via flavins secreted by *Shewanella* sp. RQs-106, when the accelerating effect of AQS on AR 18 decolorization was dominant^17^. However, in the present study, GO exhibited better catalytic behaviour than AQ. This result indicated that the bio-reduction process of GO has an important contribution to this synergistic effect. It was reported that *Shewanella oneidensis* MR-1 primarily utilizes the Mtr respiration pathway (including the proteins OmcA, MtrC, MtrA, MtrB and CymA) to reduce GO^18,19^. When the *mtrA* or *mtrB* gene was deleted, the ability of the mutants to reduce GO obviously decreased. However, additional anthraquinone-2,6-disulfonate (AQDS) significantly increased GO reduction by mutants^19^. This result indicated that the intracellular bio-reduction pathways of GO and AQDS are different. Thus, in the present study, it was assumed that AQ-GO addition not only increased the extracellular electron transfer pathway (from AQ to GO and then to 2-CA) but also broadened intracellular electron transfer pathways. The increase in electron transfer pathways resulted in the higher DHA of the bacterial consortium in the AQ-GO-mediated system (16.5 ± 0.3 mg TTF/L·h) than those in the AQ- (10.8 ± 0.4 mg TTF/L·h) and GO-mediated systems (12.5 ± 0.5 mg TTF/L·h) alone.

**Figure 4:**
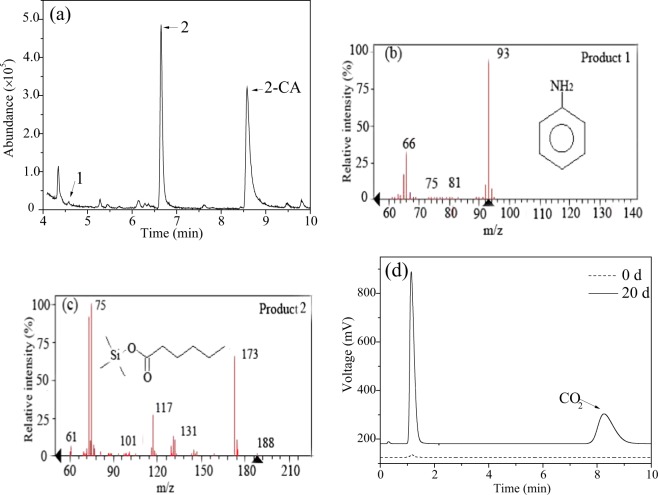
GC-MS chromatogram of the degradation products of 2-CA and GC chromatogram of CO_2_ from hexanoic acid degradation. (**a**) Total ion chromatogram; (**b**) Mass spectrum of aniline; (**c**) Mass spectrum of hexanoic acid; (**d**) GC chromatogram of CO_2_.

**Figure 5:**
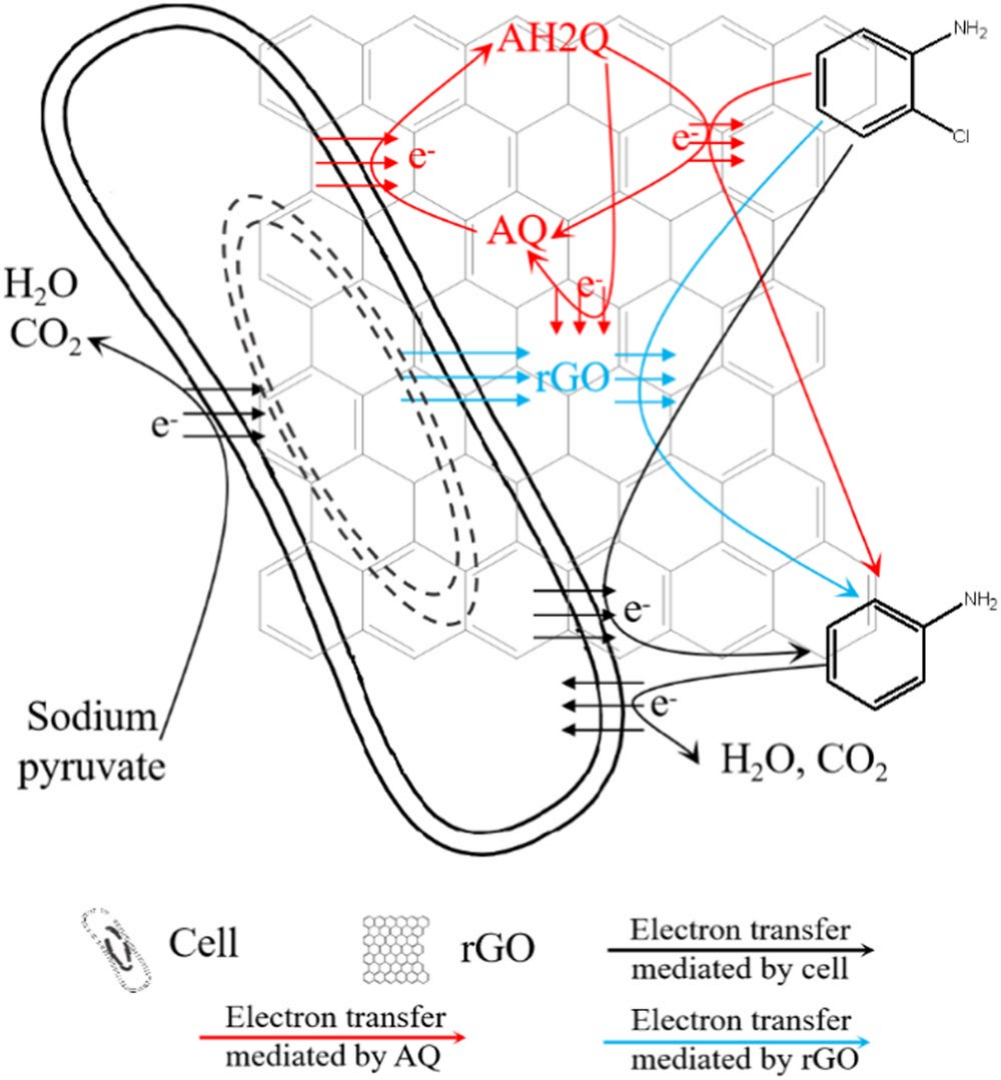
Electron transfer pathways of AQ-GO-mediated 2-CA degradation were proposed.

The 2-CA degradation products were analysed. As shown in Fig. 4, aniline and hexanoic acid were detected. As the only carbon-containing compounds in the reaction system were 2-CA and pyruvate, it was assumed that 2-CA was reduced to aniline and then transformed to hexanoic acid. This result was similar to previous studies showing that aromatic amines can undergo hydrolytic cleavage reactions^20^. Further studies have demonstrated that hexanoic acid could be mineralized to CO_2_ in 20 days when 20 mg/L hexanoic acid was used as the sole carbon source (Fig. 4). Thus, the main electron transfer pathways of AQ-GO-mediated 2-CA degradation were proposed (Fig. 5). During this process, AQ and GO were reduced by the enriched bacterial consortium, and then the formed hydroquinones and rGO transferred electrons to 2-CA. Meanwhile, the hydroquinones could also transfer electrons to GO and then to 2-CA. Moreover, AQ and rGO channelled the electrons from the dechlorination products of 2-CA, including aniline, to 2-CA, resulting in the enhanced degradation of 2-CA.

### Dynamics of the enriched bacterial consortium

To further understand the acceleration effect of the AQ-GO composite on 2-CA degradation, the changes in the structure and composition of the bacterial consortium were studied using Illumina MiSeq sequencing. After removing the incorrectly identified or poor-quality sequences, 189,876 high-quality sequences were obtained with an average length of 225 bases. The results showed (Table 1) that the number of bacterial OTUs from the AQ-GO-supplemented (CAQ) system at 35 days was 217, which is similar to that from the initial consortium (C_0_, 220) and higher than that from the control system at 35 d (CA, 204). This result indicated that the acceleration effect of AQ-GO on 2-CA degradation was beneficial for the survival of some bacteria. According to the non-parametric richness index of the Chao1 estimator, the initial bacterial consortium had the greatest richness (1081), followed by those in the CA and CAQ systems. In addition, the Shannon diversity index showed that the CAQ system had the highest diversity (2.66) among the tested three consortiums, implying that bacteria capable of reducing AQ or directly transferring electrons might be enriched. The initial consortium had a higher diversity than that in the CA system due to 2-CA toxicity. Considering that the degradation rate of 2-CA in the AQ- and GO- supplemented systems was slightly higher than that in the reaction system without mediators, the changes in the bacterial consortia in the two systems were not investigated.

**Table 1:** EPS and DHA analysis in reaction systems during 2-CA degradation.

Contents	Reaction systems	Control	AQ	GO	AQ-GO
Bound EPS (mg/L)	Proteins	45.56 ± 2.28	46.90 ± 2.34	49.78 ± 1.99	57.06 ± 1.14
	Polysaccharide	121.09 ± 2.42	139.38 ± 9.76	159.38 ± 1.60	207.05 ± 8.28
Free EPS (mg/L)	Proteins	18.48 ± 0.55	21.67 ± 1.08	24.73 ± 1.23	29.95 ± 0.60
	Polysaccharide	44.34 ± 0.89	48.06 ± 0.96	58.45 ± 2.92	75.35 ± 3.01
DHA (mg TF/L·hr)		7.49 ± 0.37	10.75 ± 0.43	12.54 ± 0.48	16.54 ± 0.33

**Figure 6:**
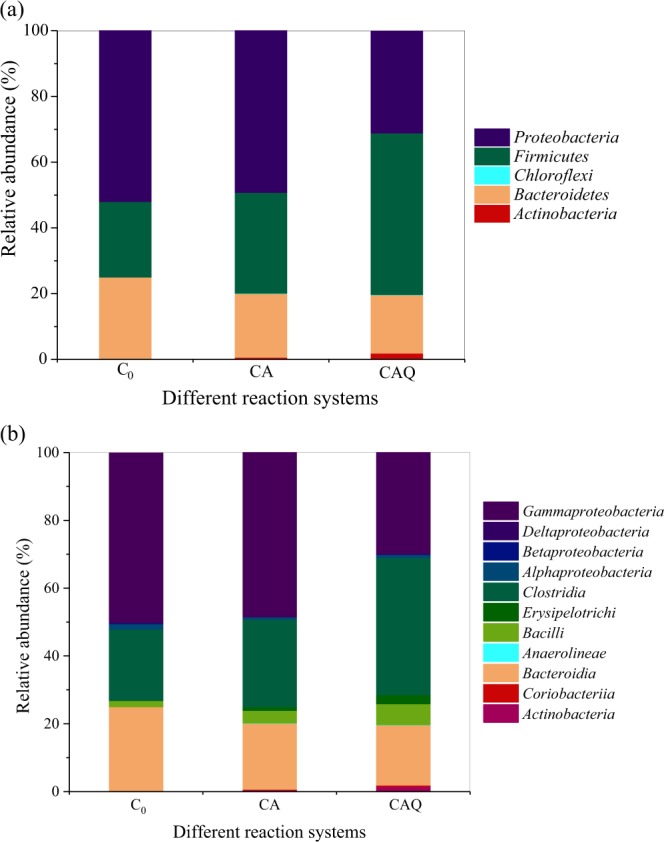
Relative abundance of species at bacterial phylum (**a**) and class (**b**) levels. C_0_, Initial bacterial consortium; CA, the bacterial consortium in the control system without AQ-GO after 35 d; CAQ, the bacterial consortium in AQ-GO supplemented system after 35 d.

These high-quality sequences were further assigned to different taxonomic levels (from phylum to genus). Figure 6 shows that *Proteobacteria*, *Bacteroidetes* and *Firmicutes* were the predominant phyla in the tested reaction systems; these phyla have been reported to be responsible for the dechlorination and degradation of chlorinated compounds^21,22^. In particular, the relative abundance of *Firmicutes* in the CAQ system (49.20%) was over 1.6-fold higher than those in the C_0_ (23.0%) and CA (30.70%) systems due to AQ-GO addition, implying that some species of *Firmicutes* played important roles in electron transfer for 2-CA degradation. Further analysis found that the increased population of *Firmicutes* was attributed to the increase in the populations of the classes *Clostridia* and *Bacilli* in the CAQ system. It has been reported that some strains of the classes *Clostridia* and *Bacilli* are capable of reducing ACs and dechlorination^23–27^. Accordingly, it was assumed that the two classes might be involved in the AQ-GO-mediated degradation of 2-CA. In contrast to *Firmicutes*, AQ-GO addition resulted in significantly decreased relative abundance of *Proteobacteria*. This result was mainly attributed to the decreased relative abundance of *Gammaproteobacteria* (48.40%, CA; 30%, CAQ), suggesting that some species of *Gammaproteobacteria* exhibit weak competitive abilities.

**Figure 7:**
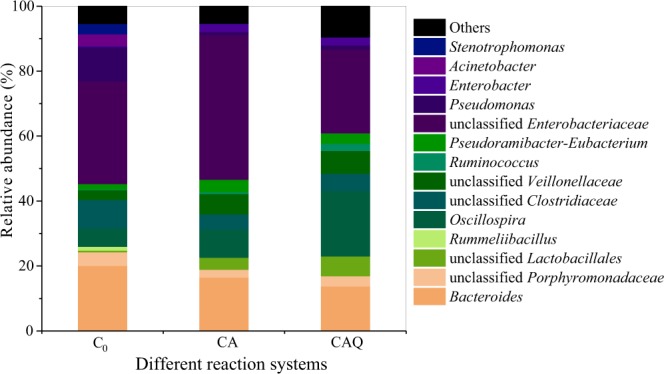
Relative abundance of species at bacterial genus level (relative abundance >1%). C_0_, Initial bacterial consortium; CA, the bacterial consortium in the control system without AQ-GO after 35 d; CAQ, the bacterial consortium in AQ-GO supplemented system after 35 d.

**Figure 8:**
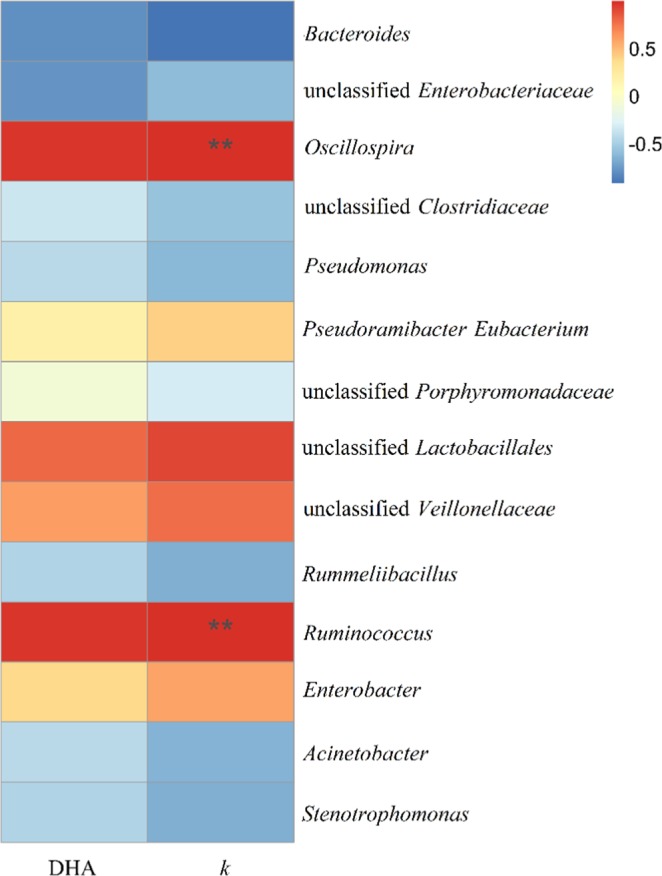
The heatmap shown the correlation between the abundance of bacteria at the genus level and two factors in the C_0_, CA and CAQ systems. Spearsman’s correlation was applied to assess the relationship between the main microorganisms and factors. **Indicates that p < 0.01. (Abbreviations: DHA, dehydrogenase activity; k, rate constant of 2-CA degradation).

At the genus level, 32 genera were detected in the C_0_, CA and CAQ systems. Figure 7 presents the tested genera with relative abundances above 1% in at least one of the tested bacterial consortia. The addition of 2-CA resulted in the increased relative abundance of some dominant genera: *Oscillospira* (5.6%, C_0_; 8.6% CA), unclassified *Lactobacillales* (0.5%, C_0_; 3.7% CA), unclassified *Veillonellaceae* (3.0%, C_0_; 6.2% CA), *Pseudoramibacter*-*Eubacterium* (1.70%, C_0_; 3.8% CA), *Enterobacter* (0.3%, C_0_; 2.5% CA) and *Ruminococcus* (0.2%, C_0_; 0.6% CA). Further analysis showed that the above these species exhibited positive correlations with the rate constant (*k*) of CA degradation (Fig. 8). Among these species, the members of *Lactobacillales* have been reported to be associated with the degradation of aromatic compounds^28^. In contrast to *Lactobacillales*, *Veillonellaceae* strains have been found to be capable of dechlorination during pentachlorophenol degradation^29,30^. The genus *Ruminococcus* has been reported to be involved in interspecies hydrogen transfer^31^. The addition of AQ-GO resulted in a significantly increased relative abundance of *Oscillospira* (belonging to *Clostridia*, 8.6%, CA, 20.2%, CAQ), Similarly, the populations of unclassified *Lactobacillales* (belonging to *Bacilli*, 6.1%), unclassified *Veillonellaceae* (belonging to *Firmicutes*, 7.0%), *Ruminococcus* (belonging to *Clostridia*, 2.2%) in the CAQ system were higher than those in the C_0_ and CA systems. Our analysis showed that these species have positive correlations with DHA of the bacterial consortium (Fig. 8), indicating that these species were involved in electron or hydrogen transfer. Notably, members of *Lactobacillales*^32^ have frequently been detected in bioelectrochemical systems, and *Veillonellaceae* family members contain many iron(III)-reducing species^33^, indicating that the two species were capable of electron transfer. Figure 8 also shows that the genera *Oscillospira* and *Ruminococcus* exhibited significantly positive correlations with the *k* and DHA, implying that they played important roles in CA degradation and electron transfer.

The present study demonstrated that the AQ-GO composite could accelerate not only the transformation of the electron acceptor 2-CA but also the degradation of aniline (dechlorination product of 2-CA). Moreover, GO as a carrier is superior to the previously reported carriers for immobilizing AQs^34–36^ owing to the electron transfer ability of rGO and the synergistic effect between rGO and AQ. Previous studies have shown that AQ-GO has a slight accelerating effect on Cr(VI) reduction by *Acinetobacter* sp. HK-1^9^. In the present study, AQ-GO significantly promoted the dechlorination and degradation of 2-CA by the enriched bacterial consortium. It seems that the catalytic performance of AQ-GO in bio-treatment systems depends on the bacterial species. Further composition analysis of the bacterial consortium showed that some species involved in the degradation of aromatic compounds and dechlorination were detected in the enriched bacterial consortium. Among these species, the relative abundances of the species capable of electron and interspecies hydrogen transfers further increased due to the presence of AQ-GO. This study showed that the suitable bacterial species play important roles in the AQ-GO-mediated degradation of 2-CA. The catalytic performance of the formed composite of AQ-GO and bacterial consortium was dependent on not only the activities of bacterial consortium, but also the electron transfer capacity of bio-reduced AQ-GO. Previous studies found that biofilm attached on AQS-PUF or activated carbon fibers partly hindered their redox mediator capacities^36,37^. When suitable internal recycle was used in AQS-PUF-mediated reactor, AQS-PUF exhibited a good catalytic performance in 75 days^36^. In this study, GO as a carrier is superior to PUF due to the electron transfer ability of rGO. Therefore, it is possible that AQ-GO has better catalytic performance than AQS-PUF in the practical application.

## Conclusion

Our study showed that an AQ-GO composite could promote the dechlorination and degradation of 2-CA and dichloroanilines by an enriched bacterial consortium. Moreover, AQ-GO exhibited better catalytic performance than AQ and GO alone. Illumina MiSeq sequencing revealed that AQ-GO addition resulted in a significantly increased relative abundance of *Firmicutes*. At the genus level, the populations of the genera *Oscillospira*, unclassified *Lactobacillales*, unclassified *Veillonellaceae* and *Ruminococcus* exhibited positive correlations with the rate constant of 2-CA degradation and the dehydrogenase activity of bacterial consortium. These findings indicated that the enrichment of functional bacteria and higher bacterial activities resulted in the enhanced dechlorination and degradation of 2-chloroaniline.

## Methods

### Chemicals

2-CA, 2,5-dichloroaniline (2,5-DCA), 2,6-dichloroaniline, 2,3-dichloroaniline, AQ and bis(trimethylsilyl)trifluoroacetamide (BSTFA, containing 1% trimethylchlorosilane) were purchased from Tokyo Chemical Industry Co., Ltd. Graphene oxide (GO) was obtained from Nano Ang Graphene Technology (Beijing, China). All other chemicals used in this study were of the highest analytical grade.

### Enrichment and cultivation of the bacterial consortium

Anaerobic sludge was collected from Dalian Bio Chemical Co., Ltd (Liaoning, China). Anaerobic sludge (approximately 5 g/L of suspended solids) and 20 mg/L 2,5-DCA were added to 135 mL serum bottles containing 100 mL deoxygenated basal medium and incubated at 30 °C under anaerobic conditions. When approximately 80% 2,5-DCA had been removed, in 70 days, 20% of the culture was transferred and subcultured under the same conditions. The process was repeatedly performed until the enriched bacterial consortium was capable of degrading various chloroanilines. The basal medium (pH 7.0) contained pyruvate 3.50 g/L, NaCl 1.00 g/L, KH_2_PO_4_ 0.20 g/L, NH_4_Cl 0.27 g/L, MgCl_2_·6H_2_O 0.41 g/L, KCl 0.52 g/L, CaCl_2_ 0.11 g/L, yeast extract 0.01 g/L, trace element solution 0.10% (v/v) and vitamin solution 0.05% (v/v)^38^. The trace element solution comprised (g/L): EDTA 15, CoCl_2_·6H_2_O 0.24, MnCl_2_·4H_2_O 0.99, CuSO_4_·5H_2_O 0.25, NaMoO_4_·2H_2_O 0.22, NiCl_2_·6H_2_O 0.19, NaSeO_4_·10H_2_O 0.21, NaWO_4_·2H_2_O 0.05, ZnSO_4_·7H_2_O 0.43 and H_3_BO_4_ 0.01.

### Preparation of the AQ-GO composite and its characterization

The AQ-GO composite was prepared as described by Zhang *et al*.^9^ Briefly, 0.10 g GO was dispersed in 100 mL deionized water by water bath sonication (53 kHz, KQ-200DB, Kunsan Ultrasonic Instrument Co., Ltd, China) for 1 h. Then, 0.2 g AQ was dissolved in 100 mL of deionized water (pH = 8–9, adjusted by adding ammonia) by water bath sonication for 2 h. Then, the aqueous dispersion of GO was added to the AQ-containing solution in a 500 mL round-bottom flask. The flask was placed in a water bath at 80 °C and stirred for 24 h. The reaction mixture was cooled to room temperature and washed with deionized water via filtration using cellulose ester dialysis membranes to remove the excess AQ. The obtained AQ-GO composite was dried for subsequent experiments. SEM (KYKY-AMRAY-1000B, USA) was used to investigate the morphologies of the AQ-GO and GO. The chemical compositional changes on the surface of the two materials were analysed using Fourier transform infrared spectroscopy (FTIR, EQUINOX55, Germany), XPS (ESCALAB 250Xi, England) and XRD with a Cu Anode (D/Max 240003030502, Japan).

### AQ-GO-mediated degradation assays of 2-chloroaniline

The AQ-GO-mediated degradation of 20 mg/L 2-chloroaniline was conducted in 135 mL serum bottles containing 100 mL of the deoxygenated basal medium described above and a certain concentration of AQ-GO (0–80 mg/L) under anaerobic conditions. After the above enriched bacterial consortium was incubated in basal medium containing 20 mg/L 2-chloroaniline for 30 days, the enriched bacterial consortium was added to the described bottles in an anaerobic incubator (YQX-II, Shanghai Xinmiao Medical Device Manufacturing Co., Ltd, China). The initial biomass was 65 mg_protein_/L. Then, these inoculated bottles were incubated at 30 °C. Moreover, in the presence of 20 mg/L AQ-GO composite, the degradation of 20 mg/L 2-chloroaniline was compared with that in the presence of 20 mg/L AQ or 20 mg/L GO. The AQ-GO-mediated degradation of 20 mg/L aniline was also studied under the same conditions. Control systems provided with the heat-killed bacterial consortium or without the bacterial consortium were also investigated. The kinetics of 2-CA degradation can be described using the following equation:

1 $${C}_{t}={C}_{0}{e}^{-kt}$$

where *k* (d^−1^) is the pseudo-first-order rate constant, *C*_0_ is the initial concentration of 2-CA and *C*_*t*_ is the concentration of 2-CA at time *t* (day). All treatments and controls were run in triplicate. Samples (1 mL each) were collected with sterile needles and syringes every 5 days for the detection of 2-chloroaniline and aniline.

The degradation products of 2-CA were analysed. Samples were collected from the reaction system with 2-CA as an electron acceptor and pyruvate as an electron donor after 20 days of incubation and then centrifuged at 10,000 g for 5 min. The obtained supernatants were collected and treated with 20 mL dichloromethane using a liquid/liquid extraction technique. The organic phase was concentrated by the evaporation of dichloromethane under a stream of nitrogen and then dried with anhydrous sodium sulfate. Finally, the treated samples were trimethylsilylated with 0.3 mL of BSTFA at 70 °C for 30 min and then analysed using a gas chromatograph-mass spectrometer (GC-MS). The anaerobic biodegradation of 20 mg/L hexanoic acid by the enriched bacterial consortium was further investigated by using hexanoic acid, an intermediate metabolite of 2-CA, as the sole carbon source. After 20 days of incubation, a 0.5 mL sample from the headspace gas in the bottles was collected and analysed for CO_2_ detection.

### Detection of EPS and DHA

After the reactions were over, samples (20 mL each) were collected from the control, AQ, GO and AQ-GO-supplemented systems, and the supernatants were collected by centrifugation (10 min at 10,000 g) at 4 °C for free EPS extraction. The harvested cells were washed twice with phosphate buffer (50 mmol/L, pH 7.0) for bound EPS extraction using the formaldehyde–NaOH method^39^. The concentration of proteins in EPS was determined using the Lowry method with bovine serum albumin as the standard. The carbohydrate content was measured by the anthrone method using glucose as a standard. Samples (20 mL each) were also collected from the AQ, GO, and AQ-GO supplemented systems, centrifuged at 10,000 g for 10 min at 4 °C and washed three times with 0.9% NaCl. The obtained cells were resuspended in 0.9% NaCl to analyse the DHAs of the bacterial consortia in different systems by using the TTC assay^40^.

### Analytical methods

The cell concentration was determined using the Lowry method. The concentrations of 2,5-DCA and 2-CA were analysed using an HPLC (SHIMADZU LC-20A, Japan) equipped with a C18 column (4.6 × 250 mm, 5 µm). The mobile phase was composed of 30% water and 70% methanol at a flow rate of 1.0 mL/min, and the detection wavelengths of 2,5-DCA and 2-CA were 254 nm. The degradation products of 2-CA were analysed using a Model 6890 gas chromatograph equipped with a Model 5975 mass spectrometer and a DB-5MS column (30 mm × 0.25 mm × 0.25 m, Agilent Technologies, CA, USA). Electron impact mass spectra were obtained at an ionization potential of 70 eV, an ion source temperature of 230 °C, an emission current of 300 μA, a mass range from 60 to 300 m/z, and a scan rate of 1s. The oven temperature was maintained at 80 °C for 2 min, increased to 120 °C at a rate of 3 °C min^−1^, and finally increased to 280 °C at a rate of 30 °C min^−1^. CO_2_ production was monitored using a gas chromatograph equipped with a thermal conductivity detector (GC-17A, Shimadzu, Japan). Helium was the carrier gas at a flow rate of 25 mL/min. A conventional three-electrode system was employed using an Ag/AgCl as a reference electrode, a platinum sheet as a counter electrode and a glassy carbon electrode (GCE) as a working electrode. Cyclic voltammetric responses of 0.5 mM potassium ferricyanide were obtained in 0.1 M H_2_SO_4_ at a scan rate of 20 mV/s when GCE, GO/GCE, AQ-GO/GCE, the mixture of AQ and GO/GCE, the bio-reduced AQ-GO/GCE were used as working electrodes, respectively. To obtain bio-reduced AQ-GO, two samples (100 mL each) were collected from 20 mg/L AQ-GO-mediated reaction systems after 10 days of incubation. Then the two samples were combined and centrifuged at 9000 × *g* for 5 min, washed with 1 M NaOH, 80% ethanol, 1 M HCl and ultrapure water three times, respectively, to remove the bacteria^41^. The washed sample was phase separated in a mixture of 1:1 N-hexane:deionized water and centrifuged at 5000 × *g* for 6 min. The black bio-reduced AQ-GO appeared at the interface of two solvent phases. Finally, the bio-reduced AQ-GO was collected in a separate glass vial, washed with deionized water and dried in vacuum desiccator at 50 °C^42^.

### DNA extraction, PCR and Illumina MiSeq sequencing

Samples (10 mL each) were collected from the control and AQ-GO-mediated systems at 0 d and 35 d. The genomic DNA was extracted using a PowerSoil^TM^ DNA Extraction Kit according to the manufacturer’s instructions. PCR amplification was carried out using the primers 520F (5′-AYTGGGYDTAAAGNG-3′) and 802R (5′-TACNVGGGTATCTAATCC-3′) for the V4 region of the 16S rRNA gene as described by Zhou *et al*.^36^ PCR products were sequenced on an Illumina MiSeq platform at Shanghai Personal Biotechnology Co., Ltd. The sequencing data were filtered and merged using Flash (version 1.2.7). The non-amplified region sequences, chimaeras, and out-target sequences were then removed using Qiime (version 1.7.0) and Mothur (version 1.31.2). Finally, the obtained valid sequences were clustered into operational taxonomic units (OTUs) according to 97% sequence similarity using Qiime. The Chao1, Simpson and Shannon indices were calculated. Sequences were phylogenetically assigned to taxonomic classifications using an RDP classifier with a confidence threshold of 80%.

The correlation between the abundance of species at bacterial genus level and the DHA/*k* were analysed by “psych” package and the result was displayed by “pheatmap” package in R.

## Supplementary information

Supporting information

## Data Availability

All data related to this experiment are provided within the article and the Supplementary Information.

## References

[1] Radianingtyas H, Robinson GK, Bull AT (2003). Characterization of a soil-derived bacterial consortium degrading 4-chloroaniline. Microbiology.

[2] Hong J, Tezel U, Tas DO, Paulostathis SG (2013). Influence of quaternary ammonium compounds on the microbial reductive dechlorination of pentachloroaniline. Water Res..

[3] Zhu L, Lin H, Qi J, Xu X (2013). Enhanced transformation and dechlorination of p-chloronitrobenzene in the combined ZVI-anaerobic sludge system. Environ. Sci. Pollut. Res. Int..

[4] Tas DO, Pavlostathis SG (2008). Effect of nitrate reduction on the microbial reductive transformation of pentachloronitrobenzene. Environ. Sci. Technol..

[5] Srivastava SK, Pionteck J (2015). Recent advances in preparation, structure, properties and applications of graphite oxide. J. Nanosci. Nanotechnol..

[6] Fu H, Zhu D (2013). Graphene oxide-facilitated reduction of nitrobenzene in sulfide-containing aqueous solutions. Environ. Sci. Technol..

[7] Colunga A, Rene Rangel-Mendez J, Celis LB, Cervantes FJ (2015). Graphene oxide as electron shuttle for increased redox conversion of contaminants under methanogenic and sulfate-reducing conditions. Bioresour. Technol..

[8] Wang J, Wang D, Liu G, Jin R, Lu H (2014). Enhanced nitrobenzene biotransformation by graphene-anaerobic sludge composite. J. Chem. Technol. Biotechnol..

[9] Zhang H, Lu H, Wang J, Zhou J, Sui M (2014). Cr(VI) reduction and Cr(III) immobilization by *Acinetobacter* sp. HK-1 with the assistance of a novel quinone/graphene oxide composite. Environ. Sci. Technol..

[10] Lu H, Zhang H, Wang J, Zhou J, Zhou Y (2014). A novel quinone/reduced graphene oxide composite as a solid-phase redox mediator for chemical and biological Acid Yellow 36 reduction. RSC Advances.

[11] Doong R, Lee C, Lien C (2014). Enhanced dechlorination of carbon tetrachloride by *Geobacter sulfurreducens* in the presence of naturally occurring quinones and ferrihydrite. Chemosphere.

[12] Aulenta F, Di Maio V, Ferri T, Majone M (2010). The humic acid analogue antraquinone-2,6-disulfonate (AQDS) serves as an electron shuttle in the electricity-driven microbial dechlorination of trichloroethene to cis-dichloroethene. Bioresour. Technol..

[13] Cervantes FJ, van der Velde S, Lettinga G, Field JA (2000). Quinones as terminal electron acceptors for anaerobic microbial oxidation of phenolic compounds. Biodegradation.

[14] Akhavan O, Ghaderi E (2010). Toxicity of graphene and graphene oxide nanowalls against bacteria. ACS Nano..

[15] Ni S, Lee P, Fessehaie A, Gao B, Sung S (2010). Enrichment and biofilm formation of Anammox bacteria in a non-woven membrane reactor. Bioresour. Technol..

[16] Ruiz ON (2011). Graphene oxide: A nonspecific enhancer of cellular growth. ACS Nano..

[17] Zhou Y (2018). Catalytic performance of quinone and graphene-modified polyurethane foam on the decolorization of azo dye Acid Red 18 by *Shewanella* sp. RQs-106. J. Hazard. Mater..

[18] Salas EC, Sun Z, Luttge A, Tour JM (2010). Reduction of graphene oxide via bacterial respiration. ACS Nano..

[19] Jiao Y (2011). Deciphering the electron transport pathway for graphene oxide reduction by *Shewanella oneidensis* MR-1. J. Bacteriol..

[20] Kuhn EP, Townsend GT, Suflita JM (1990). Effect of sulfate and organic carbon supplements on reductive dehalogenation of chloroanilines in anaerobic aquifer slurries. Appl. Environ. Microbiol..

[21] Gregorio SD, Azaizeh H, Lorenzi R (2013). Biostimulation of the autochthonous microbial community for the depletion of polychlorinated biphenyls (PCBs) in contaminated sediments. Environ. Sci. Pollut. Res..

[22] Yu H (2016). Enhanced anaerobic dechlorination of polychlorinated biphenyl in sediments by bioanode stimulation. Environ. Pollut..

[23] Martinez CM, Alvarez LH, Celis LB, Cervantes FJ (2013). Humus-reducing microorganisms and their valuable contribution in environmental processes. Appl. Microbiol. Biotechnol..

[24] Yu Z, Wang Y, Qin D, Yang G, Zhou S (2013). *Bacillus sediminis* sp. nov., isolated from an electroactive biofilm. Antonie Van Leeuwenhoek.

[25] Hobbie SN, Li X, Basen M, Stingl U, Brune A (2012). Humic substance-mediated Fe(III) reduction by a fermenting *Bacillus* strain from the alkaline gut of a humus-feeding scarab beetle larva. Syst. Appl. Microbiol..

[26] Manickam N (2014). *Bacillus mesophilum* sp. nov., strain IITR-54(T), a novel 4-chlorobiphenyl dechlorinating bacterium. Arch. Microbiol..

[27] Xu Y (2014). Enhanced abiotic and biotic contributions to dechlorination of pentachlorophenol during Fe(III) reduction by an iron-reducing bacterium *Clostridium beijerinckii* Z. Sci. Total Environ..

[28] Beer F (2017). Metabolism of foodborne heterocyclic aromatic amines by *Lactobacillus reuteri* DSM 20016. J. Agric. Food Chem..

[29] Tong H, Hu M, Li F, Liu C, Chen M (2014). Biochar enhances the microbial and chemical transformation of pentachlorophenol in paddy soil. Soil Biol. Biochem..

[30] Chen M (2016). Dynamics of the microbial community and Fe(III)-reducing and dechlorinating microorganisms in response to pentachlorophenol transformation in paddy soil. J. Hazard. Mater..

[31] Zheng Y, Kahnt J, Kwon IH, Mackie RI, Thauer RK (2014). Hydrogen formation and its regulation in ruminococcus albus: involvement of an electron-bifurcating [FeFe]-hydrogenase, of a non-electron-bifurcating [FeFe]-hydrogenase, and of a putative hydrogen-sensing [FeFe]-hydrogenase. J. Bacteriol..

[32] Masuda M, Freguia S, Wang YF, Tsujimura S, Kano K (2010). Flavins contained in yeast extract are exploited for anodic electron transfer by *Lactococcus lactis*. Bioelectrochemistry.

[33] Li H, Peng J, Weber KA, Zhu Y (2011). Phylogenetic diversity of Fe (III)-reducing microorganisms in rice paddy soil: enrichment cultures with different shortchain fatty acids as electron donors. J. Soils Sediments.

[34] Cervantes FJ, Garcia-Espinosa A, Moreno-Reynosa MA, Rangel-Mendez JR (2010). Immobilized redox mediators on anion exchange resins and their role on the reductive decolorization of azo dyes. Environ. Sci. Technol..

[35] Dai R, Chen X, Ma C, Xiang X, Li G (2016). Insoluble/immobilized redox mediators for catalyzing anaerobic bio-reduction of contaminants. Rev. Environ. Sci. Biotechnol..

[36] Zhou Y (2015). Catalytic performance of functionalized polyurethane foam on the reductive decolorization of Reactive Red K-2G in up-flow anaerobic reactor under saline conditions. Bioprocess Biosyst. Eng..

[37] Emilia R-D, Toro E, Celis LB, Cervantes FJ, Rangel-Mendez JR (2013). Enhanced microbial decolorization of methyl red with oxidized carbon fiber as redox mediator. J. Hazard. Mater..

[38] Adrian L, Manz W, Szewzyk U, Gorisch H (1998). Physiological characterization of a bacterial consortium reductively dechlorinating 1,2,3- and 1,2,4-trichlorobenzene. Appl. Environ. Microbiol..

[39] Liu H, Fang HHP (2002). Extraction of extracellular polymeric substances (EPS) of sludges. J. Biotechnol..

[40] Kang X (2011). The different metabolic activity of activated sludge samples taken from reversed A^2^/O process and conventional A^2^/O. process. Adv. Mat. Res..

[41] Shen L, Jin Z, Wang D, Wang Y, Lu Y (2018). Enhance wastewater biological treatment through the bacteria induced graphene oxide hydrogel. Chemosphere.

[42] Chouhan RS, Pandey A, Qureshi A, Ozguz V, Niazi JH (2016). Nanomaterial resistant microorganism mediated reduction of graphene oxide. Colloids Sur. B Biointerfaces.

