# Supplementary material for: Enhanced dechlorination and biodegradation of 2-chloroaniline by a 2-aminoanthraquinone-graphene oxide composite under anaerobic conditions
Source: Sci Rep. 2019 Aug 26;9:12376. doi: 10.1038/s41598-019-48904-9 (PMC6710426; doi:10.1038/s41598-019-48904-9)
Supplement: Supplementary file 1 — Supporting information [file 41598_2019_48904_MOESM1_ESM.pdf]

Supplementary Information

Enhanced dechlorination and biodegradation of 2-chloroaniline by 2-aminoanthraquinone-graphene oxide composite under anaerobic conditions

Hong Lu*, Tiantian Zhang, Yang Zhou, Jiti Zhou, Jing Wang, Xiaolei Wang

Key Laboratory of Industrial Ecology and Environmental Engineering, Ministry of Education, School of Environmental Science and Technology, Dalian University of Technology, Dalian 116024, China. Correspondence and requests for materials should be addressed to H.L. (email address: lvhonghj@163.com)

2 Results and Discussions

Following Tables and Figures complement the results already present in the main manuscript.

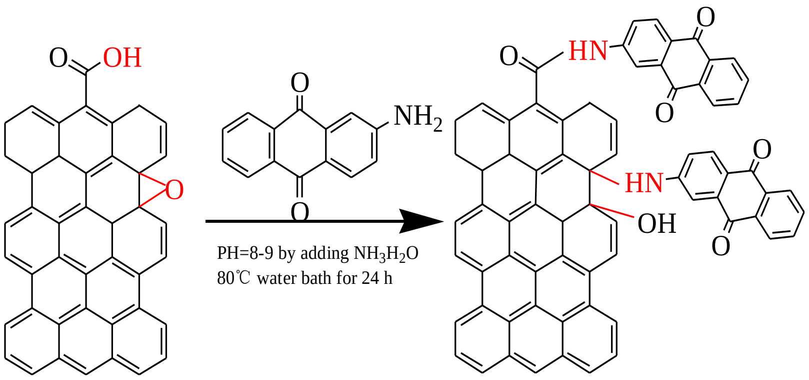

Figure 1. Schematic illustration of AQ-GO preparation

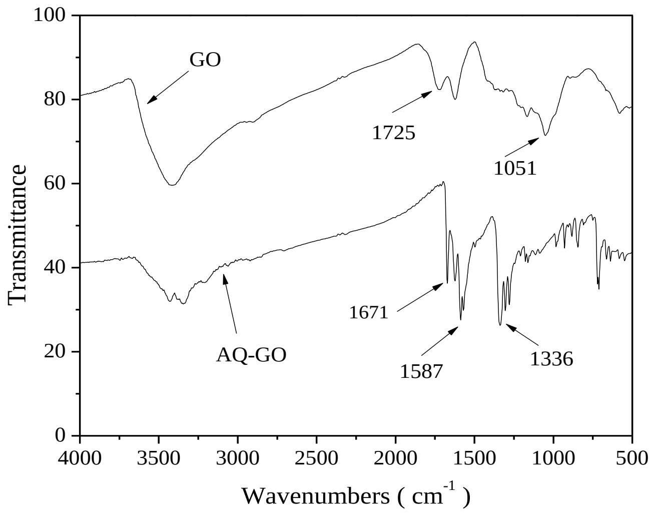

Figure 2. FTIR spectra of AQ-GO and GO

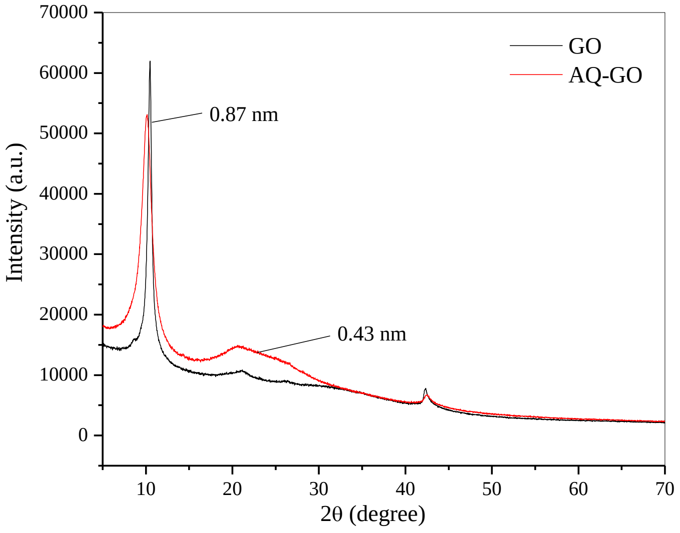

Figure 3. XRD patterns of AQ-GO and GO

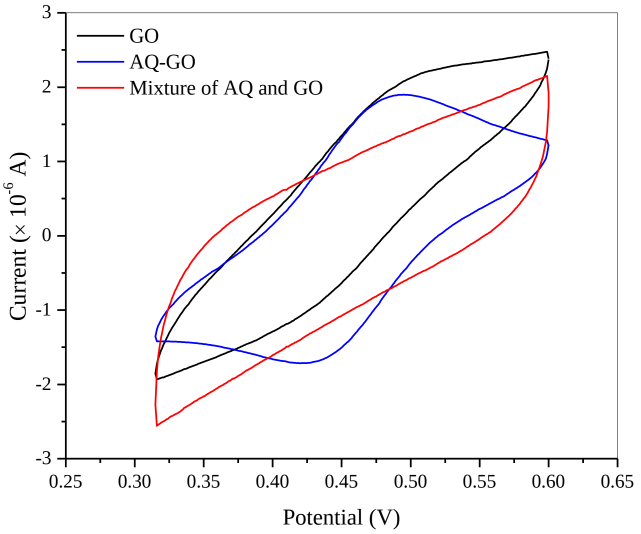

Figure 4. Cyclic voltammogram of 0.5 mM potassium ferricyanide in 0.1 M H_2SO_4 solution at a scan rate of 20 mV/s using glassy carbon electrode (GCE), GO/GCE, AQ-GO/GCE and the mixture of AQ and GO/GCE, respectively.

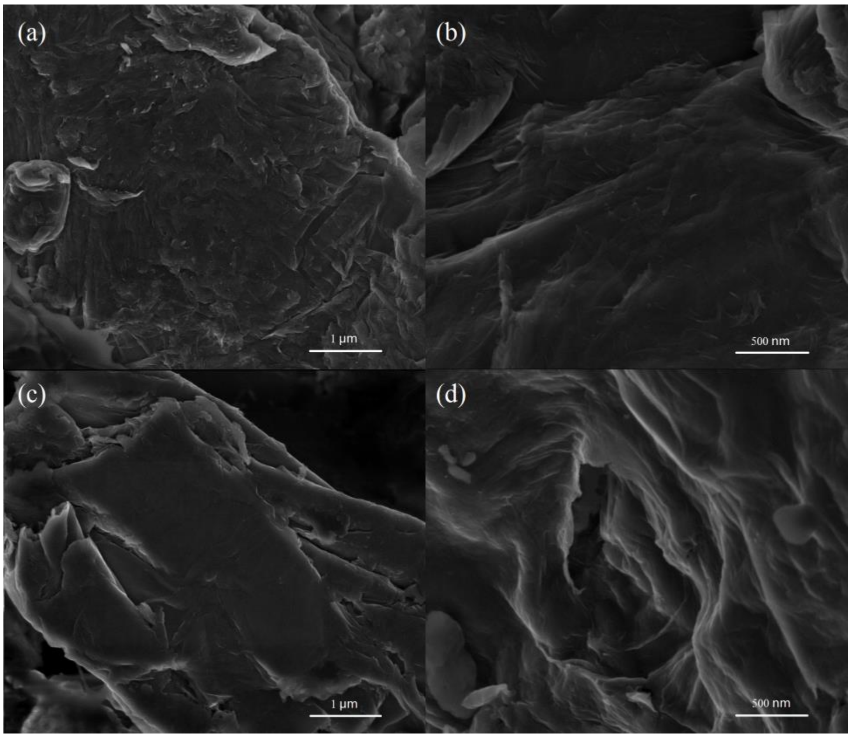

Figure 5. SEM micrographs of the surfaces and edges of GO and AQ-GO.

(a) GO surface, (b) GO edge, (c) AQ-GO surface, (d) AQ-GO edge.

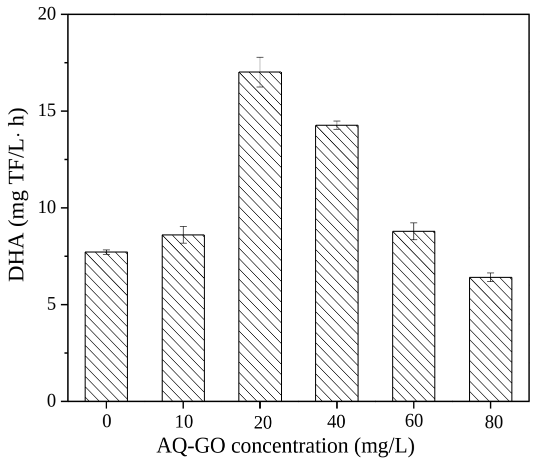

Figure 6. DHA of reaction systems with different AQ-GO concentrations. Error bars show one standard deviation.

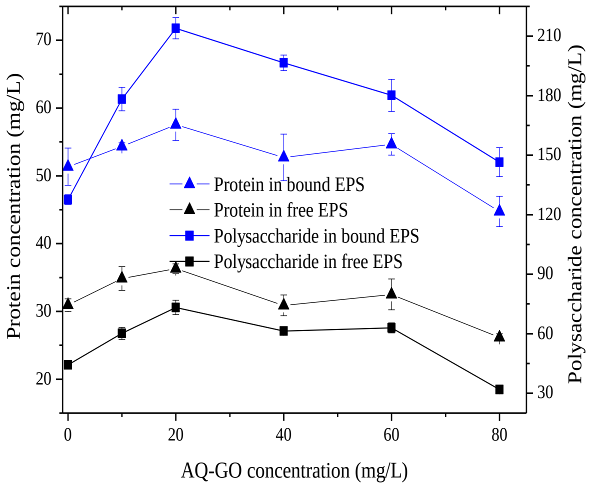

Figure 7. EPS analysis of reaction systems with different AQ-GO concentrations. Error bars show one standard deviation.

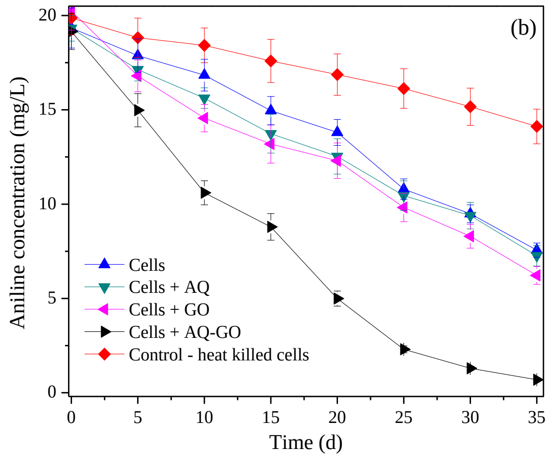

Figure 8. Effect of AQ-GO on the biodegradation of aniline.
Error bars show one standard deviation.

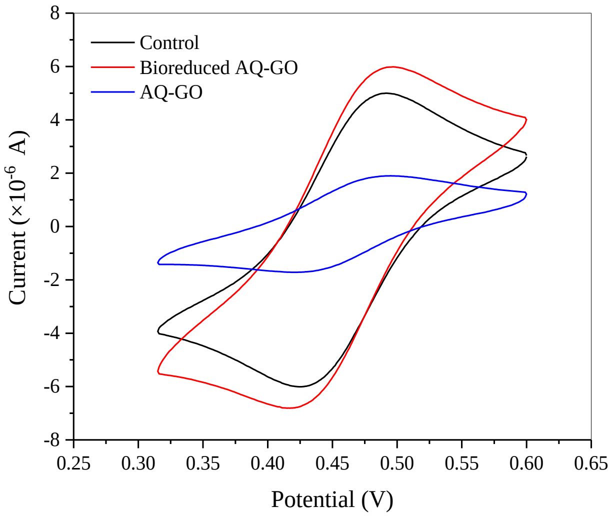

Figure 9. Cyclic voltammogram of 0.5 mM potassium ferricyanide in 0.1 M H_2SO_4 solution at a scan rate of 20 mV/s using glassy carbon electrode (GCE), AQ-GO/GCE and bioreduced AQ-GO /GCE, respectively.
